# Evaluating the Change Process of a Brief Cognitive Behavior Therapy Workshop for Psychological Distress Among Primary Care Self-Referrals in Selangor, Malaysia

**DOI:** 10.3389/fpsyt.2022.848094

**Published:** 2022-06-09

**Authors:** Jeng Mun Sam, Siti Irma Fadhilah Ismail, Kit-Aun Tan, Sherina Mohd Sidik, Zubaidah Jamil Osman

**Affiliations:** ^1^Department of Psychiatry, Faculty of Medical and Health Sciences, Universiti Putra Malaysia, Seri Kembangan, Malaysia; ^2^Department of Medicine, International Medical School, Universiti Putra Malaysia, Seri Kembangan, Malaysia

**Keywords:** depression, anxiety, primary care, Brief Cognitive Behavior Therapy Workshop, psychological distress, self-referral

## Abstract

Despite the high prevalence of psychological distress in primary care, only a limited number of individuals can benefit from early and evidenced-based psychological approach. Barriers to help-seeking attributable to stigma, lack of proper care pathway to evidence-based psychological interventions, and a high volume of primary care attendees are among the factors that contribute to the inadequate psychological treatments. This study examined the implementation of a change process using a brief cognitive behavioral therapy (b-CBT) workshop as a potential approach in managing psychological distress among 73 primary care self-referrals using purposive sampling. One-way repeated-measures multivariate analysis of variance (ANOVA) was used to analyze changes in psychological distress within a non-randomized quasi-experimental study. Results revealed significant differences in psychological distress using Depression, Anxiety, and Stress Scale-21 items at three time points (pre-intervention, post-intervention, and 1-month follow-up). Implementation of the b-CBT workshop showed positive changes for psychological distress, suggesting the possibility of integrating brief, non-stigmatized, and evidence-based psychological approach at the primary care level. However, the self-referral characteristics of the attendees remain unknown. Factors such as potential feasibility, participant's usability and satisfaction, and implementation of b-CBT workshop to improve psychological distress are discussed in this study.

## Introduction

Psychological distress is a state typically characterized by symptoms of anxiety and depression. Individuals may develop psychological distress as a response to environmental challenges, and in general, people are adaptive as the symptoms tend not to have a long-term effect on functioning (1). Most people do not require formal or specialized care and usually respond well to self-help and social support, by engaging in healthy lifestyle changes. At the primary care setting, however, psychological distress often co-occurs with somatic complaints and a range of unexplained medical problems. It is also correlated with stress and several mental disorders, has a negative impact on an individual's working capacity, personal, and interpersonal functioning [(2, 3); the World Health Organization (WHO)], and is associated with a higher number of consultations (4). Among persons with chronic diseases, psychological distress can intensify the effect of illness by increasing pain, limiting function, and decreasing adherence to treatment, which leads to poorer health outcomes and increased risks of complications (5). Findings of different studies indicate that psychological distress in the primary care setting has been prevalent in Malaysia (6–10) ranging between 6.7 and 30%. These rates are similar to studies conducted on patients seen in primary care setting in other low-income and middle-income countries (LaMICs), where at least one-third of all patients presented with psychological distress (11, 12). Unfortunately, the likelihood of primary care attendees receiving help for their psychological distress is high (13) because of a range of issues such as accessibility, level of awareness and recognition of attendees (14), accurate communication of psychological symptoms (15), and untargeted treatments (16).

While responding to the substantial impact and unmet need of psychological distress in primary care, the WHO (11, 12) has continuously informed on the integration of mental health services to primary care. Integration has the potential to improve access, respond to the needs of attendees, respect their preferences, and is safe, effective, timely, affordable, and of acceptable quality (12). In the long run, it is believed that integration can reduce stigma (17, 18), improve the provision of services, planning, and implementing strategies for prevention work (11, 17), improve awareness of treatments (19, 20), and upscale primary healthcare workers to apply psychosocial and behavioral skills (21–23). The extant literature shows that the mental health system in Malaysia has limited evidence-based and targeted interventions for primary care attendees. The targeted interventions are important to address attitudinal, awareness, and other resource-intensive factors that currently exist in dealing with psychological distress in primary care.

By bearing in mind the characteristics of psychological distress and its prevalence in primary care, there is clear potential in using cognitive behavioral therapy (CBT) workshops. Based on the studies that have been conducted in United Kingdom for mild to moderate levels of depression and anxiety (17, 21, 23, 24), CBT workshops were aimed to attract individuals who might not have proper access to primary care treatments, have poor knowledge of mental health, or faced challenges to being discriminated against and stigmatized should they seek help from specialized mental health care. As opposed to the typical weekly 10–18 sessions of CBT, these workshops were a day-long (1 day), used a self-referral route (attendees sought help without being referred), and a significant proportion of the participants were initially reluctant to seek help for their psychological distress but were receptive to the workshop format (17, 21, 25). However, challenges in conducting day-long workshops in primary care must be considered, as it is mostly not feasible for participants to commit a full working day. Situation is further exacerbated by lack of manpower; in Malaysia, there is a limited number of psychologists and counselors in primary care to conduct typical CBT intervention. In addition, due to the fast, busy, and high volume of daily attendees (26, 27), a brief, structured, and effective psychological approach is needed, which brings to the exploration of an even more condensed version of the CBT workshop.

The brief version of the CBT, the brief CBT (b-CBT), introduced by Cully and Teten (28), highlighted compressed CBT components (four to six sessions), with mild-to-moderate empirical support for treating depression (*d* = 0.33) and anxiety disorders [*d* = 1.06; (29–31)]. In two previous studies, short b-CBT workshops were conducted among primary care attendees. The first study was limited to 30 people in one group and was aimed to allow high accessibility to the public (32). The program ran from 9.30 a.m. to 4.30 p.m. and was structurally organized into four sessions. The sessional content in the study was focused on psychoeducation, behavioral activations, and identification of thoughts and challenges. In the second workshop using b-CBT as the model to construct a day-long program for insomnia among the primary care attendees, the sessions were structured and divided into seven components (33). Both studies showed that the workshops using b-CBT approach can reach out to the individuals who are reluctant to get early intervention from the general practitioners (32). High access to the workshops and satisfaction with the workshop was reported (33), and the CBT components were found effective in assisting the participants to better manage mild-to-moderate levels of psychological conditions with early interventions (32, 33).

These two studies also underscored the importance of targeted interventions for specific symptoms. For example, in the study by Horrell et al. (32), the cognitive components were targeted toward low self-confidence by identifying and challenging negative thoughts of primary care attendees. Psychoeducation was also identified as a vital component for them to understand their conditions. Then, behavioral activations and methods were used to address behavioral components related to low self-confidence, such as assertiveness in communication and problem-solving skills. According to the study by Swift et al. (33), knowledge on sleep hygiene, cognitive restructuring of the negative thoughts relating to poor sleep quality, and behavioral components of lifestyle habits affecting their sleep quality was imparted. Both studies focused on three main components in CBT, namely, psychoeducation, cognitive identification and challenging, and behavioral components (32, 33). By focusing on these three components, it is hypothetically possible to conduct a half-day, b-CBT workshop, specifically targeting those with mild-to-moderate symptoms of depression, anxiety, and stress.

According to the researchers' knowledge, a half-day b-CBT workshop for primary care attendees with psychological distress in Malaysia has not been conducted earlier. The need to use new strategies to complement the conventional way of managing mild-to-moderate levels of depression and anxiety in primary care is necessary. The b-CBT workshop approach targets issues of accessibility, financial and resource limitations, stigma, as well as mental health knowledge and recognition, which are crucial in addressing the gaps in mental health treatment. This study hypothesized that there would be a significant change in psychological distress (depression, anxiety, and stress) over pre-intervention, post-intervention, and 1-month follow-up among primary care self-referrals after receiving b-CBT intervention at primary healthcare clinics in Hulu Langat district.

## Materials and Methods

### Study Design

Three primary healthcare clinics out of 17 clinics of the equivalent sub-urban area were chosen conveniently as suggested by the head of the primary care district in Hulu Langat, state of Selangor Darul Ehsan, Malaysia. There was no control group and no randomization in this study, as there was a lack of control over variables (34), such as the number of attendees in the clinic and self-referral characteristics of the participants. All attendees who registered for the intervention were grouped as the intervention group only. Eight individual sessions were conducted in a group format. The execution of each intervention group was determined every 3 months and after the group reached a minimum of five registered attendees. A maximum of 20 registered attendees was capped for each intervention group to achieve an optimal group effect, where socialization, listening, learning, and communication are deemed essential (35).

All intervention groups used the same b-CBT workshop module and trainer. Baseline or pre-intervention measurements for all primary care attendees were obtained before the start of the b-CBT workshop to obtain homogeneity for all the dependent variables. Post-intervention measurements were administered after the intervention. Phone calls were made 1 month after each intervention in order to obtain 1-month follow-up measurements. Both post-intervention and 1-month follow-up measurements were used to assess the efficacy of b-CBT using psychological distress as the dependent variable.

A comparison was done among the three groups, namely, pre-intervention, post-intervention, and 1-month follow-up with regard to one variable, that is, psychological distress. The dependent variable was psychological distress, while the independent variables were the three time points of intervention (i.e., pre-intervention, post-intervention, and 1-month follow-up).

### Participants

A total of 73 primary care attendees (14 men and 59 women) aged above 18 years participated in the study. These participants were recruited through purposive sampling using screening instruments [Depression, Anxiety, and Stress Scale, 21 (DASS-21) items]. The participant's inclusion criteria were as follows: (1) a primary care attendee registered with either one of the three selected primary care clinics, (2) mild-to-moderate levels of psychological distress using the DASS-21 questionnaire, and (3) self-referred to the b-CBT workshop after being exposed to the promotional material (i.e., flier) of the workshop. The participant's exclusion criteria were as follows: (1) a primary care attendee registered with other primary care clinics, not selected as the study locations, (2) severe levels of psychological distress, and/or psychiatric disorder(s), and/or cognitive impairments, and (3) being referred by any healthcare professionals. The participant's flowchart using the Transparent Reporting of Evaluations with Non-randomized Designs (TREND) statement can be referred in the Supplementary Material.

### Study Procedure

The participants were screened for eligibility for the study using the sociodemographic questionnaire and DASS-21. Participants who fulfilled the inclusion criteria were given a flier to attend the b-CBT workshop and were encouraged to register themselves *via* email, a social media website, phone call, SMS, WhatsApp message, or a hardcopy registration form placed at the main reception of the primary care clinics. Reminders were sent to the participants 48 h before the b-CBT workshop.

On the day of the b-CBT workshop, the researcher team explained the research purpose and obtained written consent from the participants. The sociodemographic and DASS-21 questionnaire were completed by the participants prior to the b-CBT workshop. After the b-CBT workshop was completed, participants were required to complete the DASS-21 questionnaire post-intervention and 1-month follow-up. Participants were interviewed through a telephone call for the 1-month follow-up. Telephone interviews were selected as an option to replace physical data collection at the point of follow-up, as participants may not be able to return for a face-to-face self-administered questionnaire because of their voluntary participation. Participants were also asked a simple qualitative answer about the study for feedback regarding the quality and usability of the b-CBT workshop.

Eight face-to-face individual workshops in a group format were conducted throughout the data collection period from the year 2017 to 2018. All the interventions were conducted by the same trainer who is a professional clinical psychologist working in a national university in Malaysia as an academician and practitioner. The workshop was based on a manual adapted from the b-CBT manual by Cully and Teten (28). Each b-CBT intervention workshop ran for a duration of 4 h, including a 15-min break time.

### B-CBT Workshop Module Development

Components from the b-CBT workshop module were adapted from the b-CBT module handbook (28). The adaptation of the five components, namely, symptoms of stress, depression, and anxiety, challenging thoughts and maladaptive thought patterns, goal setting, problem-solving skills, deep breathing relaxation technique, and resource sharing was based on cognitive and behavioral theory, which is an evidence-based psychological intervention recommended by the National Institute for Health and Care Excellence (36) and the Clinical Practice Guidelines for Major Depressive Disorder and Generalized Anxiety Disorder (37–40).

The b-CBT workshop module was mainly based on having cognitive and behavioral components that are “prescriptive-based,” short, and easy for self-management. Among all the techniques in the module, the five components that were chosen for the b-CBT workshop can be replicated by any non-mental health professional who has gone through trainings as a qualifier.

The development of b-CBT workshop module started from extraction of the components from the manual by Cully and Teten (28), focus group discussion with eight panel experts (i.e., six practicing clinical psychologists, one psychiatrist, and one family medicine specialist), and a pilot study with the primary care attendees. The modules were finalized to five main topics for the half-day workshop, with the title “Stress Management Workshop” translated to Malay Language, as the national language in Malaysia. The title was chosen to reduce stigmatization to mental health treatments. For an overview of the content of b-CBT workshop, refer to Supplementary Material.

### Measurements

The DASS-21 was used in the study for screening, pre-intervention, post-intervention, and 1-month follow-up. DASS-21 is a shortened version of DASS-42, developed by the same authors, Lovibond and Lovibond (41), which is able to provide the same structure as the full version but requires half the time to complete. It has been validated with outpatient clinics in Malaysia and has been shown to be a vital research tool for gauging the incidence and relation of depression, anxiety, and stress among the population (42, 43). The internal consistency reliability using Cronbach's alpha for depression (0.84), anxiety (0.85), stress (0.84), and overall (0.94) was found satisfactory among outpatient clinic population in Malaysia (43). Therefore, the present study used the Malay version of the DASS-21 to measure the symptoms of psychological distress, which includes depression, anxiety, and stress components.

To assess sociodemographic information of the participants, self-generated items were used. Demographic data such as age, gender, marital status, ethnicity, education level, employment status, and monthly income were collected. Participants were also asked about prior mental health treatments obtained, types of treatments obtained, and self-declaration of severe mental health conditions.

## Statistical Analysis

Raw data were entered into the Statistical Package for Social Science, 25.0 version (SPSS 25.0). Descriptive statistics was used to understand the sociodemographic variables as inferential statistics. To test the research hypotheses, one-way repeated-measure MANOVA was used to analyze whether there was a difference in psychological distress scores between pre-intervention, post-intervention, and 1-month follow-up intervention. The sum of depression, anxiety, and stress scores for psychological distress was computed and tested using one-way repeated-measure multivariate analysis of variance (MANOVA) across the three time points. The analysis of variance (ANOVA) compares all the individual mean differences simultaneously to check if there was a significant difference in means in all the three dependent variables between the three time points.

Before the final analysis of the data gathered from the phase 2 study, two approaches, namely, intention-to-treat (ITT) and per-protocol analyses were considered. The ITT analysis includes all participants in the study. This analysis ignores “noncompliance, protocol deviations, withdrawal… and maintains prognostic balance” (44). The deviations from the missing data were reported on the basis of the TREND statement, which is specifically developed to guide standardized reporting of nonrandomized controlled trials (45). Missing data were dealt with using the Last Observation Carried Forward (LOCF) strategy (46). For time-series study, LOCF is chosen compared with Next Observation Carried Backward (NOCB), Linear Interpolation, and Seasonal Adjustment + Linear Interpolation. LOCF is chosen for the study as compared to other methods, as it is a method to reduce bias in analysis when data have an apparent trend (47). The rationale for using LOCF was to avoid reduction of sample size and power of the study. In addition, it could reduce a biased subset for those who continued to enroll in all the study time points, which could affect the outcome of the study. Due to the nature of the study being a psychological intervention, it is important to use LOCF by including the dropouts and use the participant's last response “carried forward” to their next response (with an idea that they did not improve), so that overestimation of the effectiveness of the intervention is controlled (47). Consequently, the ITT analysis, which is the analysis to include all the participants involved in the study, was used to complement with LOCF (48).

## Results

### Study Populations

The participants in the study were all primary healthcare attendees in the Hulu Langat district. The mean age of the participants in the sample was 45.7 years (*SD* = 8.7). Of them, 19.2% were men (*n* = 14) and 80.8% of them were women (*n* = 59), and 61.6% native Malay and 38.4% non-native Malay. Regarding the education level, 8.2% completed primary school, 24.766% completed secondary school, 2.7% completed a certificate course, 9.6% completed diploma or pre-university, and 54.8% completed bachelor's degree and above. Table 1 presents the demographic information of participants.

**Table 1:** Descriptive statistics (*n* = 73).

**Sociodemographic profile**	***F***	**Percentage (%)**	**SD**	**Range**
Age			8.6	35
18-59	73	100
Age above 60	0	0
Gender			0.39	
Male	14	19.2
Female	59	80.8
Ethnicity			0.49	
Native Malay (i.e., *Bumiputera*)	45	61.6
Non-native Malay (i.e., *Non-bumiputera*)	28	38.4
Religion			4.93	
Muslim	44	60.3
Non-muslim	29	39.7
Education level			1.51	
Primary school	6	8.2
Secondary school	18	24.7
Certification	2	2.7
Diploma or pre-university	7	9.6
Bachelor's degree and above	40	54.8
Marital status			0.59	
Married	60	82.2
Single	7	9.6
Divorced	2	2.7
Widow	4	5.5
Work status			1.78	
Work in private sector	10	13.7
Work in public sector	40	54.8
Work in semi-private sector	1	1.4
Student	12	16.4
Retired	10 13.7
Unemployed	0	0
Monthly household income level			1.53	
Below RM1000	11	15.1
RM1001 to RM2000	4	5.5
RM2001 to RM3000	9	12.3
RM3001 to RM4000	4	5.5
RM4001 and above	45	61.6
Depression			15.5	3
Mild depression	43	58.9
Moderate depression	20	41.1		
Anxiety			14.5	2
Mild anxiety	51	69.8
Moderate anxiety	22	30.2		
Stress			25.5	2
Mild stress	62	84.9
Moderate stress	11	15.1		

### Multivariate Analysis of Variance

The study has one dependent variable, namely, psychological distress (three levels—depression, anxiety, and stress). The adjusted statistical significance was *p* < *0*.01 for psychological distress.

The MANOVA produced a significant difference in psychological distress scores, *p* < 0.01; Wilks' lambda = 14.71, partial eta squared = 0.24, across time among participants who attended the b-CBT in the primary healthcare clinics in the district of Hulu Langat. The within-subjects effect showed that there is no homogeneity of dependent variables covariance matrix. Therefore, Mauchly's test of sphericity is not assumed. Huynd-Feldt correction was used, as the epsilon was >0.75 (49) (depression, ε = 0.90; anxiety, ε = 0.94; and stress, ε = 0.96). Table 2 shows the within-subject effect for time on psychological distress.

**Table 2:** Within-subject effect for time on psychological distress.

**Within-subject effect**		**F**	**Sig**.	**Partial eta squared**
Psychological distress	Wilks' lambda	14.71	0.00	0.4

Following the significant results obtained in MANOVA, each domain from the dependent variables (psychological distress: depression, anxiety, and stress) was then analyzed using the univariate tests. There was a statistically significant difference for depression scores, *F*_(2, 1.80)_ = 30.03, *p* < 0.01, partial eta squared = 0.30, anxiety scores, *F*_(2, 1.88)_ = 21.82, *p* < 0.01, partial eta squared = 0.23, and stress scores on pre-intervention, post-intervention, and 1-month follow-up, *F*_(2, 1.97)_ = 42.95, *p* < 0.01, partial eta squared = 0.37. The linear components in the within-subject contrasts for depression, anxiety, and stress showed significant results, *p* < 0.01.

Participants' scores on depression, anxiety, and stress changed across the three time points. To determine how participants' scores changed significantly over time on each subscale, Bonferroni follow-up comparisons were performed to access pairwise differences for the main effect of time. Specifically, pairwise comparisons produced a significant reduction in depression between pre-intervention and post-intervention and between pre-intervention and 1-month follow-up. Anxiety and stress produced a significant reduction between pre-intervention and post-intervention, post-intervention and 1-month follow-up, and pre-intervention and 1-month follow-up. Table 3 summarizes the mean, mean differences, and standard deviation for psychological distress at pre-, post-intervention, and 1-month follow-up.

**Table 3:** Means and standard deviations for psychological distress at pre-intervention, post-intervention, and 1-month follow-up.

**Psychological distress**	**T_**1**_**	**T_**2**_**	**T_**3**_**
	**Mean (SD)**	**Mean (SD)**	**Mean (SD)**
Depression	5.70^*^ (4.79)	3.01^*^ (3.36)	2.23^*^ (1.68)
Anxiety	5.54^*^ (3.73)	4.00^*^ (3.23)	2.38^*^ (2.33)
Stress	5.84^*^ (3.40)	3.67^*^ (3.47)	2.32^*^ (1.77)

* *p < 0.01*.

*T_1_, Pre-intervention; T_2_, Post-intervention; T_3_, 1-month follow-up*.

## Discussion

This study investigated the feasibility and practicability of a half-day b-CBT workshop in alleviating psychological distress among self-referral primary care attendees. The format, implementation strategies, location, research methodologies, and content of the workshop are discussed in this section. Results showed that there is a significant difference between pre-intervention, post-intervention, and 1-month follow-up for psychological distress after the self-referral primary care attendees attended the half-day b-CBT workshop.

### B-CBT Workshop for Psychological Distress

The results of the current study were consistent with two previous studies (32, 33) using similar CBT approach contents (i.e., psychoeducation, behavioral activations, and thoughts identification and challenging). The b-CBT workshop content from the study, adapted from Cully and Teten (28), focused on specific problem areas in psychological distress. For example, psychoeducation as the first component of the b-CBT workshop content focused on the importance of understanding their symptoms and recognizing the need to address the problem first before introducing other CBT contents. Then, behavioral activations and methods such as relaxation and problem-solving skills addressed the behavioral components of psychological distress such as the physiological state of anxiety [i.e., palpitations; (28)]. The cognitive components were targeted toward negative automatic thoughts among individuals with psychological distress that can affect their emotions and behaviors. Despite lacking measurements on the effectiveness of b-CBT workshop content, this study provided preliminary information that the content used can be important to address the symptoms of psychological distress.

The complex presentation of psychological distress among the primary care attendees can create a substantial impact leading to unmet needs among the attendees (11, 12, 16). Therefore, a targeted treatment using high accessibility and cost-effective method such as the b-CBT workshop is essential in bridging the gap between needs of psychological distress and availability of effective and practical treatments at the primary care level. The b-CBT workshop implementation, which mirrors the stepped-care approach (13, 50), allowed attendees to seek for the most effective yet least resource-intensive treatment (b-CBT workshop) before “stepping up” to a specialized tertiary healthcare system. In light of this strategy, the issue of insufficient manpower (clinical psychologist and counselor) can be addressed, as attendees with more severe distress will be seeking a more specialized one-to-one treatment.

The attendees' awareness and recognition of their psychological distress symptoms can be one of the barriers in obtaining appropriate treatments (15, 16). Accurate communication of psychological symptoms in the primary care clinics can debilitate targeted treatment, leading to a possibility of high treatment gap in the country (9, 10). By considering b-CBT workshop as one of the modalities for primary care's continuous healthcare approach to the attendees, the possibility of returning attendees owing to untargeted treatment can be improved. This can also enhance the needs of other attendees who truly need to seek physical treatments from the general practitioner, as the waiting time can be reduced after freeing up attendees who required psychological interventions.

### Applicability of B-CBT Workshop in Primary Care

The usage of b-CBT approach as an evidence-based psychological intervention at the primary care setting has indicated that the intervention was feasible in its implementation strategies. A balance between the application of evidenced-based psychological workshop content and the organization of the workshop (i.e., time, duration, and location) must be considered to facilitate participants in utilizing the self-referral route to enroll in the study. In the current study, it was found that a half-day workshop during Saturdays at the primary care facility itself was able to attract adequate participants for a group format intervention. In contrast with previous studies by Horrell et al. (32) and Swift et al. (33), which utilized a full-day workshop, the current study demonstrated a half-day workshop on a Saturday to be more appropriate for the attendees in the Malaysia, as most of them are required to commit to a full-working day.

As stated earlier, the b-CBT workshop was conducted in a group format, with <20 participants for each group. This format also followed studies conducted by Horrell et al. (32) and Swift et al. (33), with <30 participants in a group. Following the WHO's (11) recommendation on the integration of mental health services in primary care, the use of a group format can address a few factors that can be barriers to early access to care such as busy, fast, and a high volume of daily attendees (26, 27), lack of manpower in the country, and more cost-effective approaches to upscale and complement current healthcare system in Malaysia. Implementation of an evidence-based psychological intervention in a group format can provide promising results for the primary care attendees in accessing mental health services that are cost-effective and resource-intensive (22).

Despite the fact that the CBT approach originated from a western country, the results of the study indicated that the b-CBT workshop model has been well-received by the attendees. The pilot study and qualitative feedback indicated that the usage of indigenous words in Malay language (national language in Malaysia) helped them understand the terms better. For example, “jumping to conclusion” as one of the types of cognitive distortions were translated to “*membaca fikiran”* for easier understanding. In addition, some terms were directly translated using English language to Malay language's syllable. For instance, “negative automatic thoughts” were translated to “*pemikiran* (thoughts) *automatic* (automatic).”

### Research Methodologies in Primary Care

According to the studies by Brown et al. (21) and Morgan et al. (25), primary care attendees were more receptive toward the workshop format when the self-referral route was implemented. The usage of self-referral route accentuated the importance of a non-stigmatized self-initiation process, which is important to address the help-seeking barriers for individuals with psychological distress in primary care, such as stigma and discrimination. This suggests the possibility of integration of mental health services to primary care settings with appropriate implementation strategies (11, 12). Lesser participant enrolment was found in the current study compared with the studies conducted by Horrell et al. (32) and Swift et al. (33), possibly attributable to the differences in the study location, culture, and perception toward new approaches to healthcare in Malaysia. The understanding of the causes and characteristics of self-referral remained unknown in this study.

It is important to interpret the results of the study in the context of non-probability sampling and the absence of a control group owing to the implementation of the participant's self-referral route in the study. Therefore, TREND statement (45) was used in the study as the reporting guideline to describe the intervention evaluation and research design. Based on the TREND statement guideline, the b-CBT (28) was used as a framework for generating the content of the study's intervention and a quasi-experimental approach, without control group research design. This reporting statement fulfilled the development of standardized and transparent reporting for non-randomized intervention research evaluations in public health related fields.

## Limitations and Strengths

There were a few limitations in this study that should be noted. First, the scope of this study was limited to primary healthcare attendees who attended the selected primary healthcare clinics in the Hulu Langat district. The generalization to all the other urban and rural areas in the Hulu Langat district is limited. Second, the sample that self-referred to the intervention was relatively small. Individuals with mild-to-moderate levels of psychological distress may not deem it imperative to attend the intervention, thus affecting the self-referral rates. Third, there is a lack of randomization and a control group in the study, and therefore, generalization of the findings in this study should be taken with caution. The findings would be limited to the change process of the b-CBT workshop to produce an intended result without a control group among the three identified attendees of primary healthcare clinics in the Hulu Langat district. Finally, challenges in recruitment of the participants remained similar despite having a less stigmatized recruitment poster, possibly related to the self-referral route that can be new to the primary care attendees. Qualitative feedback obtained from participants who dropped out showed that their perception from the primary care attendees on the intention of running the intervention relates to marketing strategies on a specific product, requirement of payment, and doubts of the quality of the intervention.

This study has two major strengths that are worth noting. First, both the ITT and per-protocol analysis approaches were performed to confirm the examination of research hypotheses. The study used the ITT as a completer approach, which has been called for reporting from routine in the clinical psychology interventions. Second, this study is the first study in Malaysia that adopted an evidence-based psychological approach and theory-driven framework for primary care attendees. The implementation of both the b-CBT and stepped care model shed light on integrating psychological interventions to primary mental healthcare in Malaysia.

## Recommendations and Future Studies

Having the b-CBT workshop content to be manualized, this study suggested a “train the trainer” model to be applied to other non-mental healthcare professionals in the primary care clinics for the benefit of upscaling and improving accessibility of psychological interventions to the primary care attendees (23). According to the study by Joska et al. (51) and Kendrick et al. (52), nurses who are trained in psychological approaches can provide psychological assistance to the attendees, leading to a more seamless integration of mental health services as suggested by the WHO (3). This provides benefits of having a multidisciplinary healthcare system in the primary care that are easily accessible services for early interventions in the community (32).

Based on the study's result, it appeared that a “prescriptive-type psychological intervention” can be used in future studies, as the b-CBT skills are clear and easily understood. For a fast and busy primary care setting in Malaysia, short and easy-to-understand psychological interventions can offer quick problem-solving skills that can be useful for issues relating to psychological distress (6). Following the qualitative feedback indicating that self-referral to healthcare services in Malaysia requires more orientation to the approach, a directive method using evidence-based psychological approach can be potentially helpful.

Recommendations to have a longer period of study with more participants are needed for treatment randomization and a more conclusive effectiveness of the b-CBT workshop on psychological distress. Research regarding the promotion materials and methods for recruitment of self-referred attendees need to be revisited for future study replications when self-referral route is implemented. In addition, the study can use measurement tools that directly measure the effectiveness of the intervention and diagnostic tools such as Beck Anxiety Inventory and Beck Depression Inventory for depression and anxiety.

## Conclusion

This study concluded that the b-CBT workshop is a potential approach to allow change process in depression, anxiety, and stress. A psychological evidence-based approach appeared to be useful in complementing the existing healthcare system in Malaysia to address needs of the population. Potential feasibility, participant's usability and satisfaction, and implementation of b-CBT workshop to manage psychological distress can be found in this study.

## Data Availability Statement

The original contributions presented in the study are included in the article/Supplementary Material, further inquiries can be directed to the corresponding author/s.

## Ethics Statement

The studies involving human participants were reviewed and approved by Ethics Committee for Research Involving Human Subjects obtained from Universiti Putra Malaysia and Malaysia Research Ethics Committee (MREC). The patients/participants provided their written informed consent to participate in this study.

## Author Contributions

JS initiated the study with SI. The development of the intervention was done by JS and SI. K-AT contributed to designing the study and advising on the study design and data analysis. SS and ZO contributed with sharing the feasibility of the study in the primary care. All authors supported recruitment of the participants and read and approved the final manuscript.

## Funding

The authors acknowledge support from the Fundamental Research Grant Scheme (FRGS) (Reference Code: FRGS/1/2015/SS05/UPM/02/5) from Malaysia.

## Conflict of Interest

The authors declare that the research was conducted in the absence of any commercial or financial relationships that could be construed as a potential conflict of interest.

## Publisher's Note

All claims expressed in this article are solely those of the authors and do not necessarily represent those of their affiliated organizations, or those of the publisher, the editors and the reviewers. Any product that may be evaluated in this article, or claim that may be made by its manufacturer, is not guaranteed or endorsed by the publisher.
