# Supplementary figures and images for: Evaluating the Change Process of a Brief Cognitive Behavior Therapy Workshop for Psychological Distress Among Primary Care Self-Referrals in Selangor, Malaysia
Source: Front Psychiatry. 2022 Jun 9;13:848094. doi: 10.3389/fpsyt.2022.848094 (PMC9231521; doi:10.3389/fpsyt.2022.848094)

Figure 1.  
Participants Flowchart

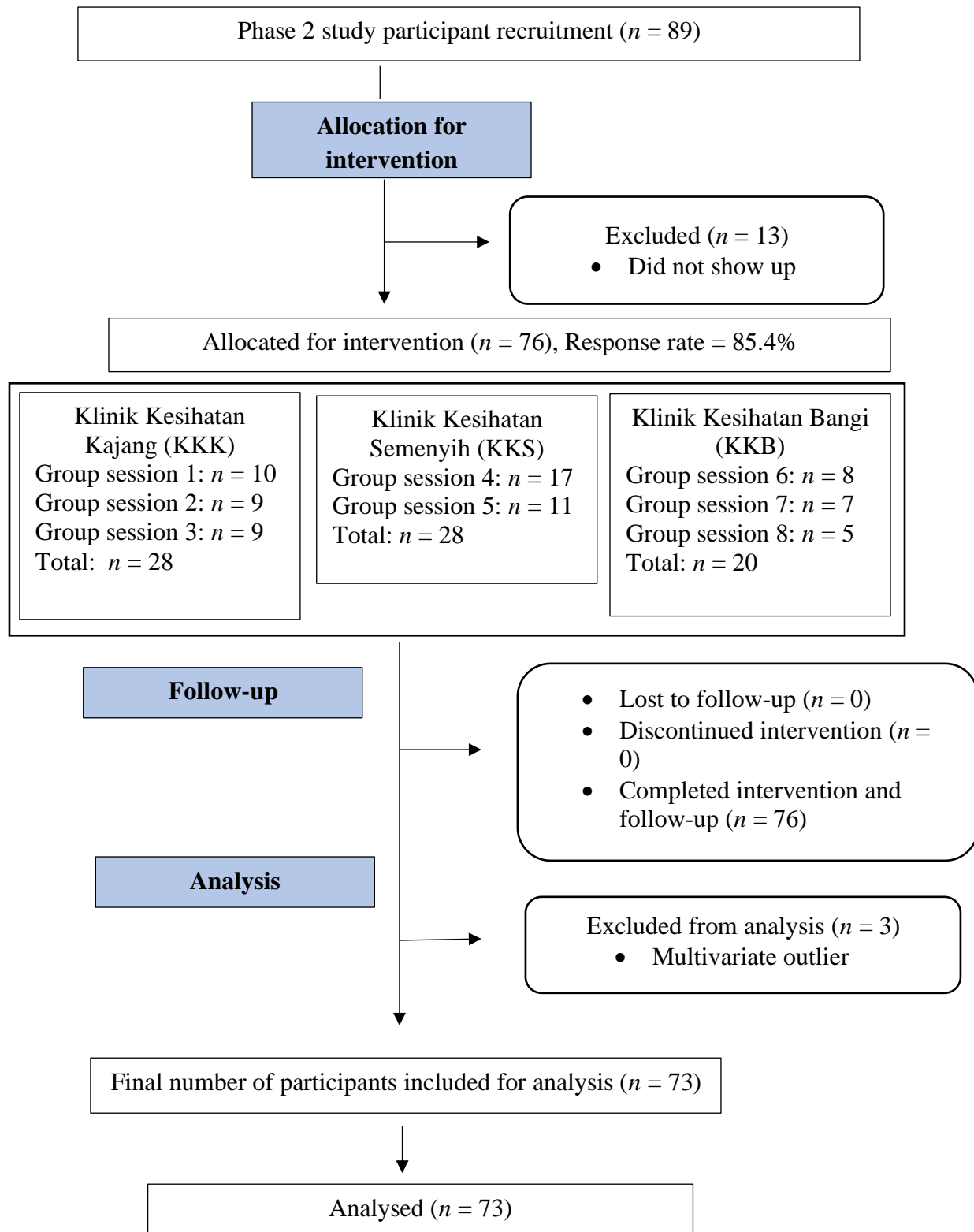

Supplement: Supplementary file 1 [file Data_Sheet_1.PDF]
