# Supplementary material for: Evaluating the Change Process of a Brief Cognitive Behavior Therapy Workshop for Psychological Distress Among Primary Care Self-Referrals in Selangor, Malaysia
Source: Front Psychiatry. 2022 Jun 9;13:848094. doi: 10.3389/fpsyt.2022.848094 (PMC9231521; doi:10.3389/fpsyt.2022.848094)
Supplement: Supplementary file 2 [file Table_1.pdf]

Table 1

Sessions Learning Outcomes

Session	Components	Sessions learning outcomes	Delivery method
1	Symptoms of depression, anxiety, and stress	• What is mental health?<br>• Prevalence of mental health in Malaysia<br>• Identify the signs and symptoms of depression, anxiety, and stress<br>• Identify the psychosocial determinants of depression and anxiety	• Didactic training<br>• Hands-on task: Handbook completion
2	Thought challenging and maladaptive thought patterns	• Understand the major components and concepts of CBT<br>• Understand the role of maladaptive thoughts and having a balanced thinking	• Didactic training<br>• Video watching<br>• Hands-on task: Handbook completion
3	Goal setting	• Understand the concept of goal setting in b-CBT<br>• Acquire skills to set feasible and appropriate goals in b-CBT	• Didactic training<br>• Hands-on task: Handbook completion

4	Problem-solving	• Using the pros and cons of engaging a behavior as problem-solving skill	• Didactic training<br>• Hands-on task: Handbook completion
5	Deep breathing relaxation	• Understand the concept and importance of relaxation in b-CBT<br>• Acquire specific relaxation skills (i.e. deep breathing relaxation)	• Didactic training<br>• Hands-on task: Skilled-based practice
6	Resource sharing	• Understand the types of mental health services that are available	• Didactic training<br>• Question-and-answer for clarifications
